# Impact of glyphosate (Roundup^TM^) on the composition and functionality of the gut microbiome

**DOI:** 10.1080/19490976.2023.2263935

**Published:** 2023-10-06

**Authors:** Lauren Walsh, Colin Hill, R. Paul Ross

**Affiliations:** a APC Microbiome Ireland, University College Cork, Cork, Ireland; b School of Microbiology, University College Cork, Cork, Ireland

**Keywords:** Glyphosate, gut microbiome, antimicrobial, human health, Roundup^TM^

## Abstract

Glyphosate, the active ingredient in the broad-spectrum herbicide Roundup^TM^, has been a topic of discussion for decades due to contradictory reports of the effect of glyphosate on human health. Glyphosate inhibits the enzyme 5-enolpyruvylshikimate-3-phosphate synthase (EPSPS) of the shikimic pathway producing aromatic amino acids in plants, a mechanism that suggests that the herbicide would not affect humans as this pathway is not found in mammals. However, numerous studies have implicated glyphosate exposure in the manifestation of a variety of disorders in the human body. This review specifically outlines the potential effect of glyphosate exposure on the composition and functionality of the gut microbiome. Evidence has been building behind the hypothesis that the composition of each individual gut microbiota significantly impacts health. For this reason, the potential of glyphosate to inhibit the growth of beneficial microbes in the gut or alter their functionality is an important topic that warrants further consideration.

## Background

Glyphosate is the active ingredient in the broad-spectrum herbicide Roundup^TM^, which was first produced in 1974. Two decades later, this facilitated the release of genetically modified crops resistant to glyphosate, or “Roundup^TM^ Ready” crops, in 1996.^1^ In 2010, glyphosate was patented as an antibiotic following decades of use as a herbicide, highlighting the compounds impressive antimicrobial activity (US Patent No. 7771736 B2).^2^ Glyphosate is one of the most popular herbicides used in the United States (US), with two hundred and eighty million pounds of glyphosate being used annually.^3^ This high level of production has resulted in the widespread presence of glyphosate in the environment through runoff and subsequent incorporation into water cycles. Glyphosate residues have been found in such places as crops (environmentally acquired and treated)^4^, drinking water,^5^ rain,^6^ animal feed^7^ and air.^8^ The herbicide is partially broken down in soil, water, and dead plant material. However, stable carbon-phosphorus bonds in the molecule protect it from complete degradation.^9^ In 2015, glyphosate was reclassified by the World Health Organization (WHO) as “probably carcinogenic to humans”.^10^ Despite this, glyphosate is still approved by the European Food Safety Authority (EFSA) and the US Environmental Protection Agency (EPA) because these carcinogenic effects are unlikely at the levels at which glyphosate residues are present in plants (0.02–19 mg/kg).^11^ However, in 2017, the European Union announced that glyphosate would only be allowed for use until December, 2022.^12^ This authorization has now been extended to December, 2023.^13^

Given the widespread presence of glyphosate in the environment, residue exposure/ingestion is a risk factor for humans with potential consequences for the human gut microbiome. The gut microbiome is home to a wide range of microorganisms and has been associated with several essential host functions including immune system development and homeostasis,^14^ modulation of energy metabolism,^15^ colonization resistance against pathogens^16^ and production of bioactive metabolites^17^. It has been connected with the functioning of other organs of the body including the skin,^18^ lungs^19^ and brain.^20^

Although the precise composition of a healthy gut microbiome is difficult to fully elucidate due to the individual variances between microbiomes and high functional redundancies between species, it has been shown that disruptions to an individual’s gut microbiome may result in disease.^21^ This occurrence is sometimes termed “dysbiosis”, in which alterations to the “normal” colonization of the gut is associated with disease.^22^ Many factors of life result in the individuality of a gut microbiome, these include such things as age, diet^23^ and lifestyle choices such as exercise frequency.^24^ Several chronic diseases from gastrointestinal to cardiovascular and metabolic diseases, to neurological and respiratory conditions also impact the gut microbiome.^25^ Inflammatory bowel disease (IBD) is one example where increases in Proteobacteria and *Bacteroidota* and a decrease in *Bacillota* have been observed in fecal samples of patients.^26^ Similarly, an increase in *Prevotella copri* and a decrease in *Bacteroides* species have been associated with the onset of rheumatoid arthritis.^26^ Samples from multiple sclerosis patients have been seen to have an increase in *Bacteroides*, *Faecalibacterium, Prevotella*, *Butyricimonas*, and *Collinsella*, with enrichment of *Bifidobacterium*, *Streptococcus, Methanobrevibacter*, and *Akkermansia muciniphila*.^27^

Glyphosate inhibits the enzyme 5-enolpyruvylshikimate-3-phosphate synthase (EPSPS) in the shikimate pathway for the production of aromatic amino acids, such as tryptophan, tyrosine or phenylalanine. This pathway is found in plants, fungi, bacteria, protozoa and archaea,^4^ rendering glyphosate an effective antimicrobial. As the human body does not produce tryptophan, tyrosine or phenylalanine, they must be acquired through diet, as well as through production by microbes in the gut.^28^ However, disruption to the shikimate pathway due to glyphosate has been shown to reduce the levels of these nutrients in plants and therefore potentially limit their bioavailability to humans who consume them.^29^ One study demonstrated that treatment with 100 µM of pure glyphosate reduced the levels of tryptophan by 13%, tyrosine by 59% and phenylalanine by 77% in sugarcane crops.^30^ Furthermore, alkaloids, which are medicinal compounds naturally produced by plants with anti-cancer and anti-inflammation properties, are also made by the shikimate pathway and so these natural compounds cannot be produced by plants following treatment with glyphosate.^29,31^

In this review, we discuss the possible effect that glyphosate has on the human body with a specific focus on the gut microbiome and the potential consequences for human health.

## Mechanisms of microbial resistance to glyphosate

**Figure 1.**
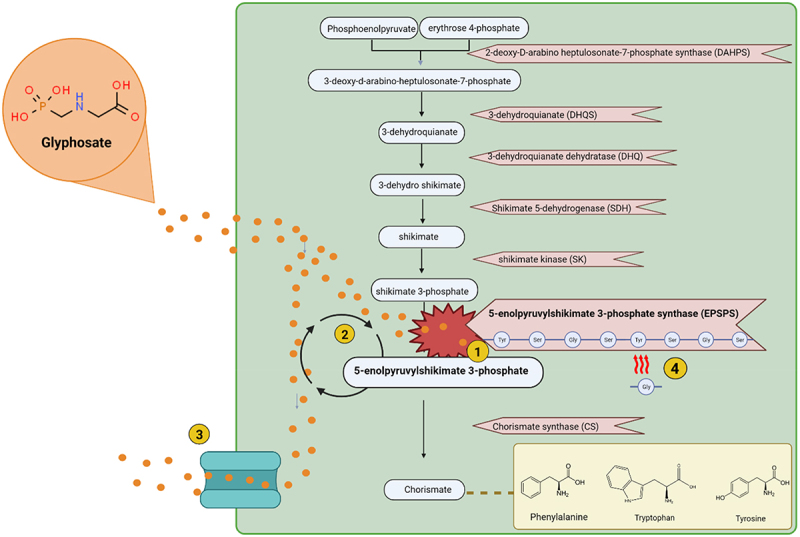
Shikimate pathway and resistance mechanisms to glyphosate. 1. Glyphosate inhibits the EPSPS enzyme. 2. The epsp gene can be overexpressed to combat EPSPS inhibition. 3. Efflux pumps are used to export glyphosate out of the cell as a form of resistance. 4. Amino acid biomarkers on the EPSPS enzyme determine the sensitivity of that enzyme to glyphosate. Changes in amino acid sequence can confer resistance to glyphosate. Created using Biorender.

The mechanism of action of glyphosate, as described in Figure 1, is to inhibit EPSPS in the shikimate pathway^4^ and is achieved by glyphosate binding to the EPSPS enzyme. The result of this is a disruption to the production of the aromatic amino acids tryptophan, tyrosine and phenylalanine.^32^

Some species of bacteria have developed mechanisms to overcome the action of glyphosate (as described in Figure 1). One such resistance mechanism is target-site resistance, in which amino acid residues found in the EPSPS active site are altered to confer resistance.^33^ Another mechanism of resistance utilized by some species is non-target site resistance, in which overexpression of the EPSPS gene occurs or efflux pumps are utilized to pump glyphosate out of the cell.^34,35^ EPSPS enzymes have been categorized into four different classes depending on their sensitivity or resistance.^36^ Class I includes sensitive versions of the enzyme, while classes II-IV describe glyphosate resistant versions. Classes I, II and IV are similar in that they are all characterized depending on the presence or absence of specific amino acid residues in the EPSPS active site. In contrast to that, class III enzymes contain a run of motifs.^37^

In general, *Bacillota* are more resistant to glyphosate when compared to *Actinomycetota* and *Pseudomonadota*. Pathogenic bacteria, such as *Escherichia coli*, *Salmonella enterica* and *Salmonella enterica* serotype Typhimurium, showed a higher level of resistance compared to host-associated commensal bacteria.^9^ One study examining the minimum inhibitory concentrations (MICs) of glyphosate (40% monoisopropylamine salt solution of glyphosate and Roundup^TM^ LB Plus) for pathogenic and commensal bacteria, found that the pathogenic strains had a significantly higher MIC (20–80 mg/mL) than the commensal strains (5–10 mg/mL).^38^ This suggests that glyphosate ingestion by humans could potentially select for pathogenic bacteria in the gut microbiome. Indeed, one study that examined 101 bacterial species for glyphosate sensitivity/resistance found that 54% of bacterial species commonly found in the gut were sensitive to glyphosate.^39^ Examples include members of the genera *Faecalibacterium*, *Citrobacter* and *Bifidobacterium*. Whereas 29% of the bacterial species evaluated were potentially resistant. Such species included members of the genera *Clostridium*, *Ruminococcus* and *Dorea*. An increase in these microbes is associated with diseases such as IBD.^40^ The remaining 17% of bacteria was made up of unclassified bacteria (10%) and bacteria varying in sensitivity (7%). Another study examined 890 bacterial strains from 101 species inhabiting the gut microbiome. They analyzed bacterial DNA sequences coding for the different types of EPSPS and predicted that 12–26% of the bacteria present in the gut microbiome are potentially sensitive to glyphosate.^33^ This result was a conservative calculation based on the strains tested to establish glyphosate sensitivity in the core gut microbiome, perhaps explaining the significant difference in results between the two studies. Some bacteria have evolved to produce enzymes which can break down glyphosate and subsequently use the glyphosate degradation products (such as phosphate) as a nutrient, an example of this is certain strains of *Pseudomonas*.^41^

A study carried out in 2020 examined the functional ability of the shikimic pathway present in bacteria using computational modeling.^42^ Fecal samples from the IBD Multi-omics Database were *in silico* screened both on a genomic and transcriptional level for indications of the presence of the shikimic pathway. The pathway was detected in 98% of 734 metagenomic samples however when the corresponding metatranscriptome samples were analyzed they found that only 35% were expressing the shikimic pathway. This suggests that in a large proportion of gut bacteria, the shikimic pathway is transcriptionally inactive and these bacteria are therefore aromatic amino acid auxotrophs. This is presumably due to the ability of gut bacteria to acquire these aromatic amino acids from the diet.

## Potential effects of glyphosate on human health

Many human studies have examined the link between glyphosate exposure and various disorders of the reproductive system,^43^ the central nervous system^44^ and the immune system.^45^ An association between glyphosate exposure and the development of non-Hodgkin’s lymphoma has been reported in multiple studies.^46–49^ However, these studies were unable to uncover a statistically significant quantitative link between glyphosate exposure and disease development. Similarly, glyphosate exposure has been linked to asthma,^50^ autism spectrum disorder^51^, and Parkinson’s disease.^44^ However, there are also studies which disprove each of these connections.^52,53^

**Figure 2.**
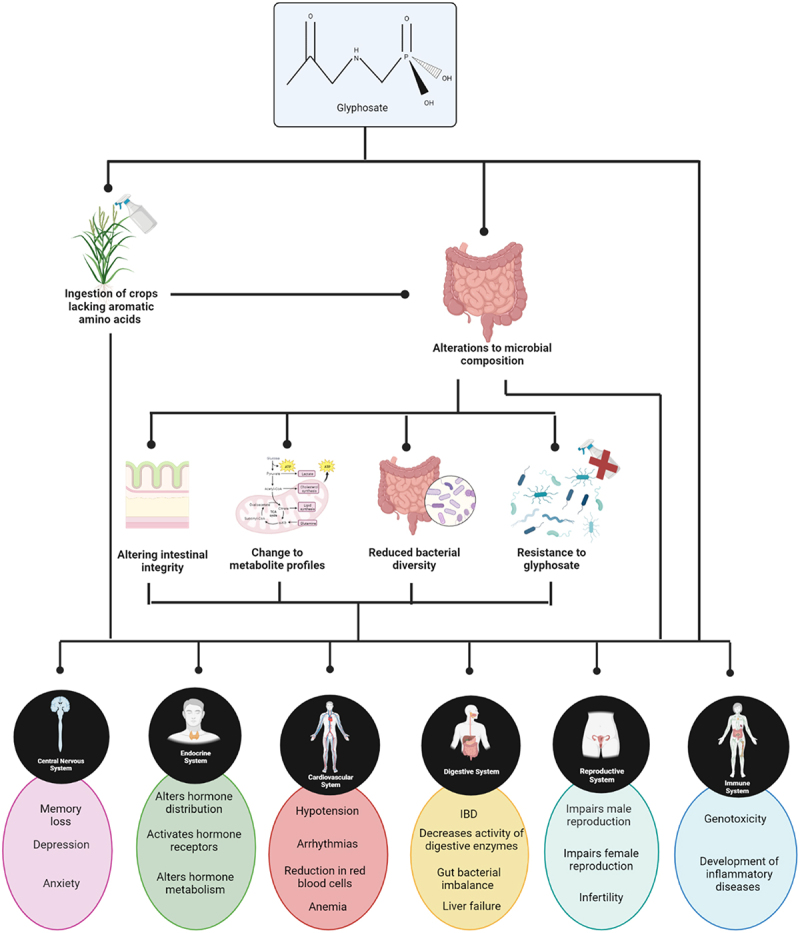
Potential effect of glyphosate exposure on the various bodily systems based on animal studies. Created using Biorender.

Information for the above-mentioned human studies was gathered via questionnaire and/or telephone interviews. This leaves room for bias in terms of participants subjectively reporting their own glyphosate exposure. In addition, participants were basing their level of glyphosate risk on their exposure to Roundup^TM^, in terms of both application of the herbicide and proximity to a family member who used the herbicide. While glyphosate is the active ingredient in Roundup^TM^, the commercial product does contain a number of other chemicals including surfactants which can themselves cause harm independently of glyphosate. There are no human studies to our knowledge with definitively known concentrations of glyphosate exposure. One exception is a study carried out by Zoller et al.^54^ where a known concentration of glyphosate (196.8 μg) and aminomethylphosphonic acid (AMPA) (1.67 μg) was fed to participants in a “test dish”. Following which, urine output was examined for glyphosate and AMPA concentrations. However, even under these circumstances conclusive concentrations of glyphosate and AMPA are hard to determine, due to the variability of the presence of glyphosate in the diet of participants. Although animal models may not always predict the outcome of human studies, they can be a useful source of information. Animal models have been used to show a link between glyphosate and the development of disorders such as obesity.^55^ diabetes,^56^ autism,^57^ and mental health disorders.^58^ In animals, glyphosate has been reported to negatively impact the gut,^59^ the cardiovascular system^60^ the endocrine system,^61^ the reproductive system,^62^ the central nervous system,^63^ and immune system^64^ (outlined in Figure 2). However these studies did not examine the effect of glyphosate on the gut microbiome of their selected animal model. Therefore, in these instances it is impossible to determine if alterations to the gut microbiome were an impacting factor in disease manifestation. It is also worth mentioning the difference between the gut microbiome of humans and animal models and how for this reason results obtained in animal models may not reflect results in humans.

A large portion of tryptophan utilized by the host is acquired through diet, however gut bacteria also play a role in the production of tryptophan for host utilization. As glyphosate has been shown to reduce microbially produced tryptophan, this suggests that glyphosate in turn reduces tryptophan availability in the host. Tryptophan and its derivatives have been studied for their role as signaling molecules connecting both the gut microbiota and host cells, subsequently affecting human health and disease.^65^ There is evidence showing an association between reduced tryptophan levels in the serum of IBD patients, as well as an increased severity of IBD symptoms.^66^ In addition, tryptophan metabolism results in aryl hydrocarbon receptor agonists which reduce central nervous system inflammation, a cause of multiple sclerosis symptoms, in a mouse model.^67^ Other properties of tryptophan derivatives include anti-inflammatory,^68^ increased immune response and barrier function.^69,70^ Although these disease consequences cannot be directly linked to glyphosate exposure, the large amount of literature linking glyphosate exposure to a reduction in microbially-produced tryptophan suggests this may be a possibility.

## Glyphosate and the gut microbiome

As previously discussed, glyphosate is present in the environment due to widespread contamination. This has ultimately led to glyphosate being unknowingly consumed by humans through means of contaminated crops and drinking water, as well as the inhalation of contaminated air.^71^ Glyphosate was present at concentrations of up to 233 ppb in the urine of 90% of farmers in a study carried out in South Carolina.^72^ In the USA, 60–95% of the general public have the compound present in their urine at concentrations of 2–3 μg/L, with the same being said for 40–50% of Europeans at concentrations of <1 μg/L.^71^ Another study examining the effect of glyphosate on kidney function in young children and infants showed that 30% of neonates examined had glyphosate present in their urine at concentrations of <1.06 μg/L.^73^ This could have occurred as a result of glyphosate present in the breast milk or baby formula,^74^ or due to the ability of glyphosate to traverse the placenta,^75^ The neonates had a higher incidence of glyphosate presence in their urine compared to 7.6% of participants in the cohort of infants aged 10–19 months. All of the glyphosate concentrations reported in the above studies do not exceed 5% of the acceptable daily intake (ADI) which is 0.5 mg/kg BW/day in the EU (EFSA),^13^.

### Impact of glyphosate on the gut microbiome composition and functionality

**Figure 3.**
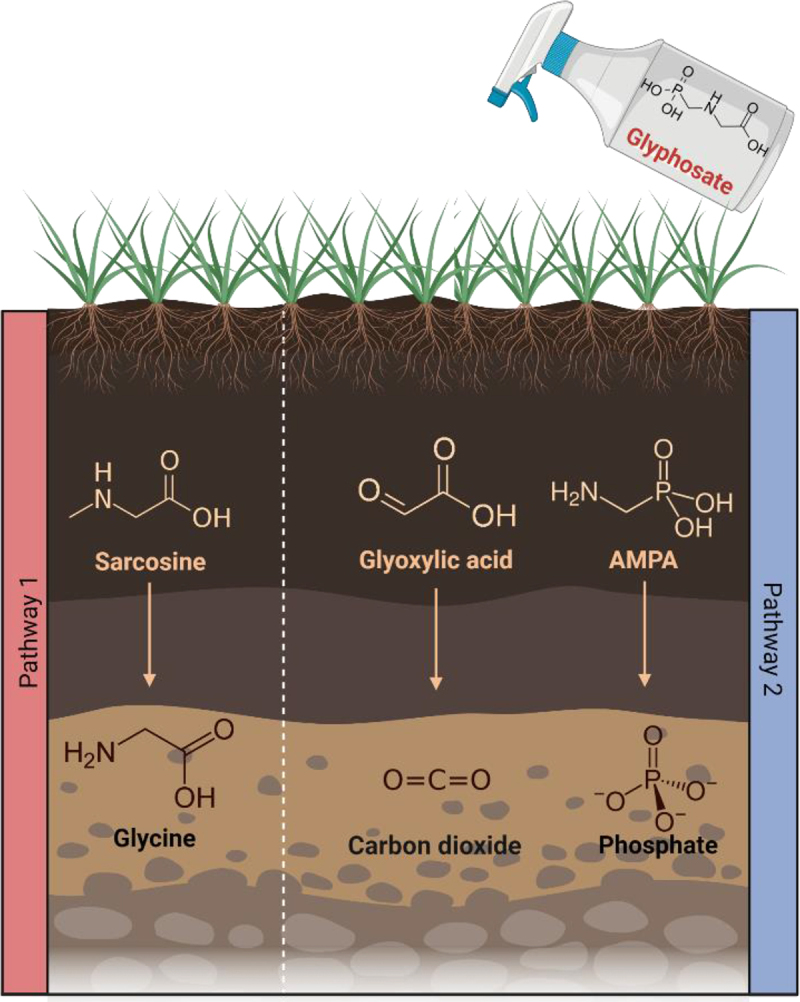
Glyphosate degradation pathways and the subsequent products resulting. Created using Biorender.

Glyphosate can be degraded in two different ways as described in Figure 3. The first pathway results in an accumulation of sarcosine, after which the glyphosate C-P bond is cleaved through dephosphorylation resulting in the formation of glycine.^76^ The second pathway involves the cleavage of the C-N bond via the enzyme glyphosate oxidoreductase resulting in the production of glyoxylate and AMPA.^77^ Various organisms in nature can degrade glyphosate, including numerous bacteria such as *Arthrobacter* spp. GLP-1, *Geobacillus caldoxylosilyticus* T20, *Pseudomonas* spp., *Rhizobium* spp., *Bacillus megaterium* 2BLW and *Alcaligenes* spp.^78,79^ Considering the ability of certain bacteria to degrade glyphosate in nature, the possibility that bacteria in the gut microbiome degrade glyphosate has been hypothesized. One study examined glyphosate degradation in the human fecal microbiota by anaerobically incubating pure glyphosate (Sigma-Aldrich) with fecal suspensions from 15 participants.^80^ The presence of AMPA was not detected following incubation and there was no evidence of degradation of glyphosate by the bacteria present in the fecal samples. This suggests that the human gut microbiome is unable to catabolize glyphosate, and as a result glyphosate is excreted, therefore limiting the potential for bioaccumulation of the compound. Sarcosine, glycine and glyoxylic acid have been shown to be present in urine.^81–84^ However, this was not demonstrated as part of a study examining degradation of glyphosate and therefore does not provide a conclusion as to how glyphosate metabolites are excreted from the body. Although one study did determine that 1% of all glyphosate (glyphosate, CAS Bioflow® 1071-83-6) initially ingested will be excreted in urine, with 23% of AMPA (AMPA, CAS Pro® 1066-51-9) being excreted in urine.^54^

Information regarding the effect of these metabolites on the host are somewhat limited, with their effect on the gut microbiome even more so. However, there have been studies performed comparing the effect of pure glyphosate (Interchim SS-7701) on the host with its metabolite AMPA (Sigma-Aldrich 324,817), which has shown that AMPA does not have a significant impact on the gut microbiome. This is in contrast to glyphosate which was shown in this study to decrease *S. alvi* five to 13 fold more than in the untreated control.^85^ The presence of AMPA in urine samples has also been associated with breast cancer patients, with one study reporting a 38% increase of samples containing AMPA in the breast cancer group compared to healthy controls.^81^ In a chronic kidney disease rat model, serum levels of glycine conjugated compounds were increased in the untreated group representative of chronic kidney disease, insinuating high levels of glycine are associated with chronic kidney disease. This occurrence was also noted to coincide with an increase in *Clostridium*, *Enterorhabdus, Parasutterella*, *Blautia*, and *Escherichia Shigella*.^86^ One study has shown that the presence of glyoxylic acid is of benefit to the host, promoting myogenesis, reducing muscle atrophy, and subsequently metabolizing to amino acid in muscle cells. Although, this study has acknowledged that more *in vivo* experiments are necessary to fully elucidate to what degree glyoxylic acid may aid the host in this respect.^87^ An increase of sarcosine is associated with the development of prostate cancer,^88^ so much so that numerous diagnostic tests have been researched utilizing sarcosine as a biomarker for the disease.^89^ In contrast, sarcosine has also been used to benefit the host by means of treating schizophrenia, with impressive outcomes when used in conjunction with antipsychotic drugs.^90^ This area would be worth researching further, as although there is information available outlining the effect these metabolites have on the host, information regarding the effect of these metabolites on the gut microbiome and related biological effects is scarce, and in need of examination.

An increase in the pH of feces has been strongly correlated to the amount of glyphosate (Bioflow®) in the colon.^91^ Similarly, an increase in glyphosate levels has a negative effect on acetate levels in the cecum.^91^ This suggests that glyphosate has a disruptive effect on acetate-producing bacteria. Glyphosate has also been shown to affect digestive enzymes such as trypsin, lipase and amylase;^92^ it is hypothesized that glyphosate inhibits lipase in digesting fats, and trypsin in digesting proteins. This causes intact proteins to enter the colon at which point they are broken down releasing ammonia, subsequently increasing the pH of the colon.

Bifidobacteria are recognized as important members of the human gut microbiota, with many strains proven to provide health-promoting properties.^93^ Bifidobacteriales is the most abundant bacterial class in the infant gut where its members are associated with infant development and health.^94^ Some bifidobacteria can metabolize non-digestible carbohydrates such as mucins and human milk oligosaccharides found in breast milk, resulting in the production of short-chain fatty acids (SCFAs) which exert important physiological functions on the host.^95^ However, bifidobacteria are known to be among the most sensitive bacteria to glyphosate.^96^ Furthermore, it has been reported that most infant formulas are contaminated with glyphosate. One study reported levels between 0.03 mg kg^−1^ and 1.08 mg kg^−1^.^97^ This could potentially further exacerbate the problem of *Bifidobacterium* reduction in the infant gut.^98^

### Potential health consequences of glyphosate-associated microbiome alterations based on animal studies

**Figure 4.**
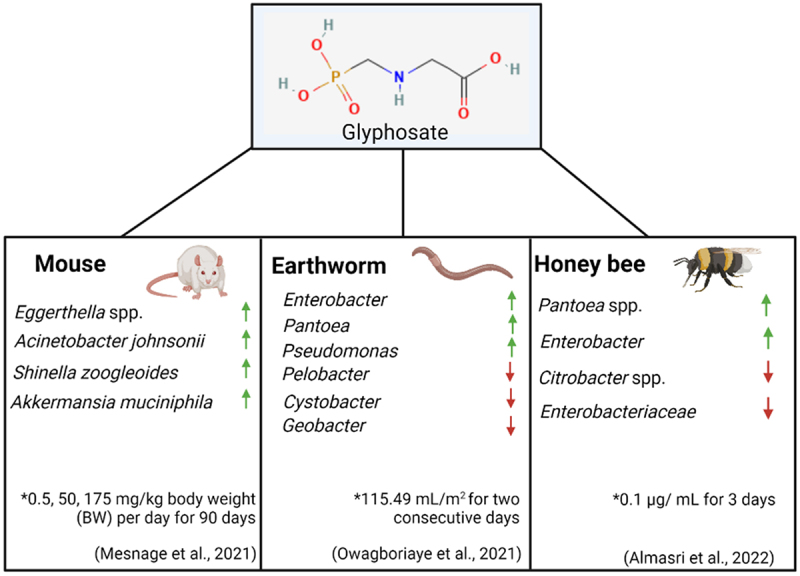
Examples of animal models used to examine the effect of glyphosate on the gut microbiome and the increase and decrease in bacterial species present. * - concentrations of glyphosate used and duration of experiment. Created using Biorender.

In recent years a variety of studies in animals have examined the effects of glyphosate on the gut microbiome, as seen in Figure 4. Although it should be noted concentrations of glyphosate used in these studies which resulted in biological consequences are between 3.5 and 500 fold higher than the ADI. These studies have revealed that glyphosate-associated alterations to the gut microbiome are linked to disturbances in a variety of systems around the body. For example, gut dysbiosis resulting from glyphosate exposure has been shown to contribute to male reproductive toxicity in rats.^99^ Specifically, male rats that ingested feed containing glyphosate (GLY, *N*-(phosphonomethyl)-glycine, purity > 95%) at levels of 250 mg/kg BW for two months were seen to have a reduction in sperm motility, an increase in sperm malformation and impairments to testes structure. 16S rRNA sequencing of the gut microbiome of the rats showed significant changes to the commensal bacterial population. An increase in *Prevotella* and *Bacteroides* was observed in the rats that exhibited reduced sperm quality. These findings support the hypothesis that glyphosate-induced gut dysbiosis may lead to male infertility.

The cecum microbiota of rats has been reported to be altered by glyphosate exposure (0.5, 5, 50 mg/kg body weight/day) (Pure glyphosate, Roundup^TM^ Bioflow® and Ranger Pro®). In this case, Bacteroidota were decreased, while Bacillota and Actinomycetota were increased.^100^ In addition to changes to the composition of the gut microbiota following glyphosate exposure, alterations to intestinal integrity have also been reported in adult male mice. This was confirmed by changes to the circulation of syndecan-1 proteoglycan and the expression of ZO-1 and ZO-2 tight junction effector proteins. In this particular study, Bacteroidota, Pseudomonadota and Desulfobacterota relative levels were altered as a result of glyphosate (Round Up^TM^) exposure from pregnancy until adulthood at a concentration of 0.075% *w/v* present in the drinking water.^101^

Homocysteine is a metabolite which can be converted into cysteine in the presence of specific B vitamins. Deficiencies in vitamins B12, B6 and folic acid have been shown to induce hyperhomocysteinemia (a condition where there is more than 15 μmol/L of homocysteine in the blood). One study examining the effect of pure glyphosate and Roundup® in male pups (1.75 mg/kg bw/day for 49 days), determined that the compound induced hyperhomocysteinemia.^60^ This is thought to be due to glyphosate reducing the levels of *Prevotella*, which can biosynthesize B vitamins. This is a conflicting report to that of Liu et al.^99^ mentioned above, as in that case glyphosate (GLY, *N*-(phosphonomethyl)-glycine, purity > 95%) treatment increased levels of *Prevotella*. The difference in results could be due to changes to the dosing, with a reduction in *Prevotella* abundance associated with a much lower treatment of glyphosate compared to the previously mentioned study. Health issues such as brain damage, memory and cognitive decline and autism have been associated with an increase in homocysteine.^60,102^ Therefore, it is hypothesized that early exposure to glyphosate may be a contributing factor in the development of such disorders.

Glyphosate has also been heavily correlated with neurodevelopmental disturbances such as autism and encephalopathy (as reviewed by Barnett et al.^103^). This is potentially due to alterations across the gut-brain axis. The ability of glyphosate to reduce the growth of commensal bacteria such as *Ruminococcaceae* spp., *Bifidobacterium* spp. and *Lactobacillus* spp. probably results in reduced levels of microbial metabolites. These metabolites, including L-glutamate which behaves as a neurotransmitter, and SCFAs involved in neuromodulation, traverse the gut-brain axis. The significance of a reduction in L-glutamate is that it is a precursor in synthesizing GABA, reductions of which are associated with neuropsychiatric disease states, such as autism.^104^ However, it is worth noting L-glutamate is also readily available in food and so is more commonly taken up through diet. This amino acid is also involved in carbohydrate metabolism in the host.^105^ As previously mentioned, some bacteria can be resistant to glyphosate including *Pseudomonas, Arthrobacter* spp. and *Geobacillus* spp. These bacteria may increase levels of reactive oxygen species and pro-inflammatory cytokines, which may lead to hypothalamic-pituitary-adrenocortical (HPA) activation and increased glucocorticoids with potential effects on neurodevelopment.^106^ Children with autism have been shown to have increased levels of clostridia compared to children who do not have autism.^107^ An association between encephalopathy and toxic metabolites produced by clostridia has also been uncovered.^108^ Clostridia species are more resistant to glyphosate than other bacterial taxa such as bifidobacteria and lactobacilli. This correlation has led to the theory that glyphosate activity on the gut microbiome is leading to changes causing a buildup of toxic metabolites which contribute to brain damage.^96^

A variety of other animal models have been commonly used to examine the effects of glyphosate on the gut microbiome, including honeybees and earthworms. Three species of earthworms were examined for disturbances to the gut bacterial community following Roundup® (7.20 g/l glyphosate) exposure at a concentration of 115.49 mL/m^2^ for two consecutive days. The abundance of *Pseudomonadota* significantly increased to become the dominant phylum.^109^ The impact of glyphosate on honeybees has been the topic of much research given their significant role in pollination. Honeybees chronically exposed to sublethal doses of pure glyphosate (10 mg/L) (active ingredient, Sigma-Aldrich) and infected with *Nosema ceranae* (a pathogenic bacterium found in adult bees) for seven days resulted in an altered immune response and inability to resist infection.^110^ Indeed, the composition of the honeybee microbiome was transformed following glyphosate exposure and *N. ceranae* infection. Specifically, the abundance of *Lactobacillus apis* and *Snodgrassella alvi* decreased, which is a significant observation considering that these bacterial species are core members of the honeybee gut microbiome. Another study examined the effect of pure glyphosate on honeybees at a concentration of 0.1 µg/L (for 3 days). This is more reflective of a concentration that is found naturally in honey. In this case, the honey bee microbiome showed a decrease of only non-core bacterial species.^111^ It should be noted that this study used a significantly reduced concentration of glyphosate compared to the previously mentioned study.

Only a few studies have utilized advanced molecular profiling to examine the effects of glyphosate on the gut microbiome. To address this knowledge gap, Mesnage et al.^112^ performed metabolomic and metagenomic sequencing on cecum samples from rats who had been exposed to both glyphosate and MON 52276, a liquid formulation of glyphosate, at concentrations of 0.5, 50, 175 mg/kg body weight (BW) per day for 90 days. As expected, metabolomic studies confirmed that the shikimate pathway in the cecum microbiome of the rat is inhibited by glyphosate.^100^ With regards to diversity in the gut microbiome, *Acinetobacter johnsonii* and *Eggerthella* spp. were increased following glyphosate treatment. While the health implications of increased *Acinetobacter* remain unknown, some members of *Eggerthella* spp. such as *Eggerthella lenta* have been linked to GI tract infections.^113^ The specific strain of *Eggerthella* that was increased in the rats used in this study has been associated with liver cirrhosis in humans.^114^ A previous study spanning over two years was carried out by the same group examining rats following an ultra-low dose of daily Roundup^TM^ (50 ng/L) exposure.^115^ The rats, in this case, developed nonalcoholic fatty liver disease, further corroborating the hypothesis that glyphosate at low levels negatively impacts the liver.

Rumen models have also been utilized to examine the degree to which glyphosate impacts bacterial communities. One such study, carried out by Ackermann et al.,^116^ used glyphosate ((*N*-phosphonomethyl) glycine), at final concentrations of 0, 1, 10, and 100 µg/ml, to treat a rumen fluid fermentation model. Glyphosate was also associated with reductions in specific groups of the microbial population, while increasing certain potentially pathogenic bacteria, however these results were not significant. Riede et al.^117^ observed minimal alterations caused by glyphosate, in the formultion of Plantaclean^R^ 360 (486 g glyphosate isopropylamine salt per Liter), on a RUSITEC ruminal fermentation. Similarly, minor variations in bacterial community were observed, however there was no indications that glyphosate exposure specifically targeted beneficial commensal bacteria or favored the growth of pathogenic bacteria. Nielsen et al.^91^ examined the effect of 5–80 mg/mL of Bioflow® 450 PLUS (450 g/L glyphosate acid equivalent) on a sheep rumen fermentation models. These results were also consistent with the previously mentioned studies, in that minimal antimicrobial activity was observed. However, this result was dependant on an environment with sufficient levels of aromatic amino acids.^91^

### Impact of the consumption of glyphosate-treated crops on the gut microbiome

As previously mentioned, the presence of glyphosate residues in crops and livestock feeds has been widely reported. While in past years glyphosate was primarily used as a means of weed control before the seeding of crops, the development of Roundup^TM^-ready crops has allowed for the use of glyphosate all year round, as the herbicide can be sprayed directly on growing plants. This development has increased the likelihood of glyphosate residues being present in crops for human consumption and crops destined for livestock feed. Monsanto has reported that glyphosate is of minimal risk to mammals and so feed and crops containing glyphosate residues should be of no concern.^118^ One study that investigated the ruminal microbiome of cows following ingestion of glyphosate-contaminated (73.8 and 84.5 mg/day) feedstuffs for 16 weeks determined there were no adverse effects on the composition of the ruminal microbiome. Preparation of the feedstuff involved treatment with glyphosate pre-harvest.^119^

## Conclusion

Since its release in 1974, glyphosate has increasingly become a part of the environment, particularly with the release of the Roundup^TM^-ready resistant crops in 1996. This ultimately has led to increased ingestion of glyphosate by humans and livestock through drinking water, crops and inhalation of contaminated air. The widespread use of Roundup^TM^ is correlated with an increase in various disorders affecting almost every aspect of human physiology. It is important to acknowledge this is a correlation, not causation. Other than an increase in studies highlighting the negative effects of glyphosate exposure, there is no study to our knowledge explicitly linking glyphosate distribution to this increase in the prevalence of human disorders.

Animal studies to date convincingly suggest that glyphosate can impact a variety of critical systems in the body. However, research in humans is somewhat contradictory, with some studies indicating a link between glyphosate and cancer development, increase in asthma severity and arthritis, while other studies claim glyphosate has no impact on these disorders. To our knowledge, all human studies examining glyphosate and its interactions with the human body rely solely on self-reporting glyphosate exposure via questionnaire or telephone interview. Given this subjective means of collecting information, the data available on glyphosate exposure in human studies remain somewhat limited. There is a large bank of knowledge on glyphosate exposure in animal models. These studies have shown that glyphosate administered at the ADI that EFSA has determined safe (0.5 mg/kg BW/day) can cause harm. This was seen by Séralini et al.^120^ who observed kidney and liver dysfunction in rats following exposure to glyphosate below the ADI. However, it is unknown how these studies would translate to humans.

In terms of the gut microbiome, alterations following glyphosate exposure include increases in *Bacteroides* spp. and a decrease in *Bifidobacterium* spp., *Ruminococcus* spp., and *Lactobacillus* spp. These changes to the gut microbiome are associated with biological effects such as hyperhomocysteinemia, reproductive toxicity, alterations to intestinal integrity, neurodevelopmental disturbances, and buildup of toxic metabolites. However, the concentrations of glyphosate used in these studies were above the ADI. An example of a study which used the EFSA-determined ADI to treat rats reported an increase in *Eggerthella, A. johnsonii*, and *A. muciniphila.*^112^ There were no physiological consequences observed following glyphosate treatment at this concentration. This suggests glyphosate administered at the ADI appears not to cause microbiome disturbances significant enough to result in biological consequences, at least under the precise conditions of this trial. The absence of significant disturbances to the gut microbiome at these concentrations is most likely due to the resistance of these bacteria to glyphosate. Although reports on the sensitivity or resistance of gut bacteria to glyphosate are somewhat contradictory when the focus is on the class of EPSPS gene present, analysis of metadata suggesting that the shikimic pathway is not transcriptionally operational in a large number of gut bacteria is more convincing. This could be due to the environment of the gut providing sufficient levels of aromatic amino acids to the bacteria, therefore minimizing the need for the gut microbiota to produce these aromatic amino acids. As previously mentioned, diet can provide the aromatic amino acids necessary for both host cellular functions as well as function of the bacteria present in the host, provided the diet consumed has not already been depleted of aromatic amino acids as a result of glyphosate exposure.

Although the ADI of glyphosate determined by EFSA is a concentration high enough to cause harm in animal models, it seems unlikely that this is due to disturbances in the gut microbiome. However, there is a significant gap in glyphosate research on humans. Controlled studies treating with known concentrations of glyphosate would be required. While this may not be feasible in terms of ethics, with a lack of such research it is difficult to fully elucidate the consequences glyphosate has on both the gut microbiome and human health in general. EFSA has announced that glyphosate will not be authorized for use in the EU after December of 2023. Nonetheless, associations between glyphosate levels in humans and gut microbiome signatures should be defined. Lastly, knowledge of the impact of glyphosate exposure on microbiome functionality is essential.
